# Gβγ subunits inhibit Epac-induced melanoma cell migration

**DOI:** 10.1186/1471-2407-11-256

**Published:** 2011-06-17

**Authors:** Erdene Baljinnyam, Masanari Umemura, Mariana S De Lorenzo, Lai-Hua Xie, Martha Nowycky, Mizuka Iwatsubo, Suzie Chen, James S Goydos, Kousaku Iwatsubo

**Affiliations:** 1 Department of Cell Biology and Molecular Medicine, New Jersey Medical School-University of Medicine and Dentistry of New Jersey, 185 South Orange Avenue, Newark, 07103, USA; 2 Department of Pharmacology and Physiology; New Jersey Medical School-University of Medicine and Dentistry of New Jersey, 185 South Orange Avenue, Newark, 07103, USA; 3 Department of Chemical Biology, Susan Lehman Cullen Laboratory of Cancer Research in the Ernest Mario School of Pharmacy, Rutgers University, 60 Frelinghuysen Road, Piscataway, New Jersey, 08854, USA; 4 Division of Surgical Oncology, Department of Surgery, UMDNJ-Robert Wood Johnson Medical School, 195 Little Albany Street, New Brunswick, New Jersey, 08854, USA; 5 Cardiovascular Research Institute, Yokohama City University Graduate School of Medicine, 3-9 Fukuura, Yokohama, 236-0004, Japan

## Abstract

**Background:**

Recently we reported that activation of Epac1, an exchange protein activated by cAMP, increases melanoma cell migration via Ca ^2+ ^release from the endoplasmic reticulum (ER). G-protein βγ subunits (Gβγ) are known to act as an independent signaling molecule upon activation of G-protein coupled receptor. However, the role of Gβγ in cell migration and Ca ^2+ ^signaling in melanoma has not been well studied. Here we report that there is crosstalk of Ca ^2+ ^signaling between Gβγ and Epac in melanoma, which plays a role in regulation of cell migration.

**Methods:**

SK-Mel-2 cells, a human metastatic melanoma cell line, were mainly used in this study. Intracellular Ca ^2+ ^was measured with Fluo-4AM fluorescent dyes. Cell migration was examined using the Boyden chambers.

**Results:**

The effect of Gβγ on Epac-induced cell migration was first examined. Epac-induced cell migration was inhibited by mSIRK, a Gβγ -activating peptide, but not its inactive analog, L9A, in SK-Mel-2 cells. Guanosine 5', α-β-methylene triphosphate (Gp(CH2)pp), a constitutively active GTP analogue that activates Gβγ, also inhibited Epac-induced cell migration. In addition, co-overexpression of β1 and γ2, which is the major combination of Gβγ, inhibited Epac1-induced cell migration. By contrast, when the C-terminus of β adrenergic receptor kinase (βARK-CT), an endogenous inhibitor for Gβγ, was overexpressed, mSIRK's inhibitory effect on Epac-induced cell migration was negated, suggesting the specificity of mSIRK for Gβγ. We next examined the effect of mSIRK on Epac-induced Ca ^2+ ^response. When cells were pretreated with mSIRK, but not with L9A, 8-(4-Methoxyphenylthio)-2'-O-methyladenosine-3',5'-cyclic monophosphate (8-pMeOPT), an Epac-specific agonist, failed to increase Ca ^2+ ^signal. Co-overexpression of β1 and γ2 subunits inhibited 8-pMeOPT-induced Ca ^2+ ^elevation. Inhibition of Gβγ with βARK-CT or guanosine 5'-O-(2-thiodiphosphate) (GDPβS), a GDP analogue that inactivates Gβγ, restored 8-pMeOPT-induced Ca ^2+ ^elevation even in the presence of mSIRK. These data suggested that Gβγ inhibits Epac-induced Ca ^2+ ^elevation. Subsequently, the mechanism by which Gβγ inhibits Epac-induced Ca ^2+ ^elevation was explored. mSIRK activates Ca ^2+ ^influx from the extracellular space. In addition, W-5, an inhibitor of calmodulin, abolished mSIRK's inhibitory effects on Epac-induced Ca ^2+ ^elevation, and cell migration. These data suggest that, the mSIRK-induced Ca ^2+ ^from the extracellular space inhibits the Epac-induced Ca ^2+ ^release from the ER, resulting suppression of cell migration.

**Conclusion:**

We found the cross talk of Ca ^2+ ^signaling between Gβγ and Epac, which plays a major role in melanoma cell migration.

## Background

Melanoma causes the majority of skin cancer related death, and is prevalent worldwide. The median life span of patients with advanced stage melanoma is less than a year because no therapies are effective once the tumor has spread to vital organs [1]. The tumor metastasis process is conventionally understood as the migration of individual cells that detach from the primary tumor, enter lymphatic vessels or the bloodstream, attach to endothelial cells and undergo transendothelial extravasation, and proliferate in organs [2]. Although numerous efforts have been focused on understanding of melanoma progression, the controlling of melanoma cell migration/metastasis has been unsuccessful.

G protein-coupled receptors (GPCRs) belong to a large family of transmembrane receptors. Upon ligand binding, the G-protein α and βγ subunits (Gα and Gβγ, respectively) are dissociated. Each molecule regulates intracellular signal transductions and evokes cellular responses including cell migration [3]. Previous reports suggested a role of Gβγ in cell migration of endothelial cells and breast cancer cells [4-6]; however, the role of Gβγ in melanoma is largely unknown. Gβγ is also known to regulate Ca ^2+ ^homeostasis via regulation of membrane voltage-dependent Ca ^2+ ^channels in excitable cells [7,8]. In non-excitable cells, Gβγ activates Ca ^2+ ^release from the endoplasmic reticulum (ER) [9,10]. However, the role of Gβγ in Ca ^2+ ^signaling in cancer cells, including melanoma, remains unknown.

In addition to the traditional target of cAMP, protein kinase A (PKA), a new, PKA-independent signaling pathway has been identified. The exchange protein directly activated by cAMP (Epac), a guanine nucleotide exchange factor [11], has two isoforms, Epac1 and Epac2. Epacs mediate cAMP signaling through activation of a small-molecular-weight G protein, Rap1 [12]. Previous reports demonstrated functions of Epac in cancer cells. Epac mediates cell adhesion in Ovcar3 cells [13], apoptosis and growth arrest [14] in B lymphoma cells, formation of embryonic vasculogenic networks in melanoma cells [16], and proliferation of prostate carcinoma cells [15]. Previously, we have reported that Epac increases melanoma cell migration by modification of heparan sulfate, a major component of the extracellular matrix [17]. More recently, we demonstrated that Epac increases cytosolic Ca ^2+ ^in melanoma cells, which also led to an increase of cell migration. The major mechanism in Epac-induced Ca ^2+ ^elevation was activation of inositol triphosphate (IP3) receptor to release Ca ^2+ ^from the ER [18]. Although calmodulin is a major regulator of IP_3 _receptors [19-21], the role of calmodulin in melanoma is still unclear.

In the present study, we demonstrated that there is interaction between Ca ^2+ ^signaling between Gβγ and Epac in melanoma. The interaction evoked by Gβγ inhibited Epac-induced Ca ^2+ ^elevation and cell migration *via *additional Ca ^2+ ^influx from the extracellular space. We thus propose that Gβγ-signaling inhibits the Epac-induced cell migration via a Ca ^2+^-dependant mechanism in melanoma.

## Methods

### Reagents and cell lines

Reagents were purchased from Sigma unless otherwise specified. G-protein βγ activating peptide, myr-SIRKALNILGYPDYD-OH (mSIRK), and its control peptide, myr-SIRKALNIAGYPDYD-OH (L9A), 2-aminoethoxydiphenyl borate (2-APB), xestopongin C, ryanodine, N-(6-aminohexyl)-1-naphthalenesulfonamide (W-5) were purchased from EMD Chemicals. Guanosine 5', α-,β-methylene, triphosphate (Gp(CH2)pp) was purchased from Biomol International. 8-(4-Methoxyphenylthio)-2'-O-methyladenosine-3',5'-cyclic monophosphate (8-pMeOPT) was purchased from Biolog. Antibodies against Epac1, Gβ1, Gγ2, C-terminal domain of β-adrenergic receptor kinase (βARK-CT) were purchased from Santa Cruz Biotechnology. Anti-α-tubulin antibody was purchased from Abcam. SK-Mel-2 and SK-Mel-24 melanoma cell lines were obtained from the American Type Culture Collection. SK-Mel-187 cell line was kindly provided by Dr. Alan Houghton. C8161 cell line was provided by Dr. Mary JC Hendrix. WM1552C, WM115, WM3248, WM1361A were obtained from Dr. Meenhard Herlyn. SK-Mel-2 and SK-Mel-24 cells were maintained in MEM containing 10% FBS, 1% penicillin-streptomycin. All other melanoma cells were maintained in RPMI containing 10% FBS, 1% penicillin-streptomycin.

### Fluorescence imaging of intracellular Ca ^2+^

Measurement of intracellular Ca ^2+ ^release was performed as we previously described [18]. Cells were incubated with HEPES buffer containing 4 μmol/L of Fluo-4AM followed by washing and incubation with HEPES buffered saline containing 1.8 mmol/L of CaCl_2_. An iXon+ 885 charge-coupled device camera (Andor Technology) was used to monitor fluorescence changes. Full images were collected every 4 seconds. The field was illuminated by two wavelengths in rapid succession. Fluo-4 was excited at 488 nm, and data were expressed as normalized changes in background-corrected fluorescence emission (F/F_0_). Data were analyzed using Imaging Workbench (INDEC BioSystems). Representative Ca ^2+ ^signals averaged from 5 individual cells were shown in the figures.

### Western blot analysis

Western blot analysis was performed as we previously described [22,23]. Briefly, cells were lysed and sonicated in lysis buffer. Equal amounts of protein were subjected to SDS-PAGE. After protein separation by electrophoresis, samples were transferred to Millipore Immobilon-P membrane and immunoblotting with antibodies was performed.

### Migration assay

Migration assay was performed using the 24-well Boyden chambers (8 μm pores, BD Biosciences) as we previously described [17]. The cells were plated at a density of 1 × 10^6 ^cells/100 μl of medium in the inserts, and incubated for 3 h at 37°C followed by staining using the Diff-Quick kit (Dade Behring). Pictures were taken with a microscope and migrated cells were counted with Image J software using 10 randomly chosen fields.

### Overexpression of Epac1 or βARK-CT

Adenoviral overexpression of Epac1, βARK-CT or LacZ was performed as we previously described [18]. βARK-CT adenovirus was kindly provided by Dr. Koch. The recombinant vector was introduced into human embryonic kidney cells (HEK-293) to recover infectious adenovirus. Cells were infected with adenovirus for 24 h followed by confirmation of overexpression of target proteins.

### Overexpression of Gβ1 and Gγ2 subunits

Plasmid constructs harboring Gβ1 or Gγ2 subunit were kindly provided by Dr. Simonds [24]. Plasmid transfection was performed as we previously described [22,23]. Briefly, 750 μl of serum- and antibiotics-free OPTI-MEM was mixed with both 13.5 μl of Lipofectamine2000 and 6.25 μl plasmid DNA. The mixture was then diluted with 5 ml MEM supplement with 5% FBS and gently overlaid onto the cells, followed by incubation for 16 h. The overexpression was confirmed by western blot analyses, and the transfected cells were used for cell migration or cytosolic Ca ^2+ ^measurement assays.

### Data analysis and statistics

Statistical comparisons among groups were performed using one factor ANOVA with Bonferroni post hoc test. Statistical significance was set at the 0.05 level.

## Results

### Gβγ inhibits Epac-induced cell migration in melanoma

**Figure 1:**
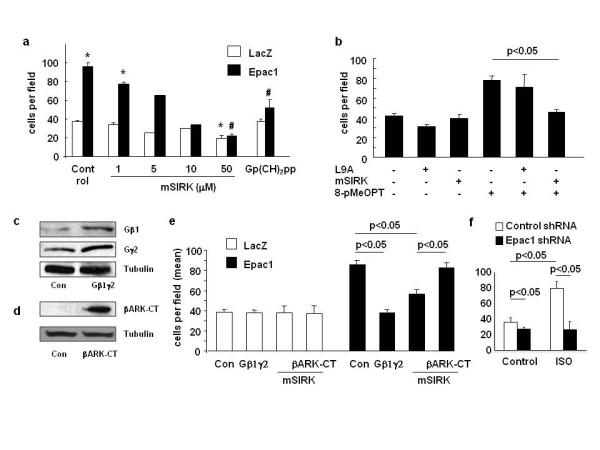
**Gβγ inhibits Epac-induced cell migration**. a) SK-Mel-2 cells were infected with adenovirus harboring LacZ or Epac1 followed by the migration assay in the presence of mSIRK or Gp(CH2)pp (10 μM). mSIRK inhibited Epac1-induced melanoma cell migration. *, p < 0.05 vs. LacZ control, #, p < 0.05 vs. Epac1 control. n = 4 except 5 and 10 μM of mSIRK (n = 2). b) Migration assay was performed in SK-Mel-2 cells in the presence or absence of 8-pMeOPT (200 μM), L9A (20 μM) or mSIRK (20 μM). mSIRK, but not L9A, inhibited 8-pMeOPT-induced cell migration. n = 4. c and d) Following the termination of adenovirus harboring LacZ or Epac1, SK-Mel-2 cells were subjected to co-overexpression of Gβ1 and Gγ2 subunits or βARK-CT. Western blot analyses showed increased expression of the target proteins. e) Migration assay was performed in SK-Mel-2 cells. Co-overexpression of Gβ1 and Gγ2 inhibited Epac1-induced cell migration. Overexpression of βARK-CT restored Epac1-induced cell migration even in the presence of mSIRK (20 μM). f) Ablation of Epac1 inhibits GPCR-induced cell migration in melanoma. SK-Mel-2 cells were infected with lentivirus harboring Epac1- or control-shRNA as we previously described [18]. Migration assay was performed in the presence or absence of isoproterenol (ISO) (100 μM), a β-adrenergic receptor agonist. Ablation of Epac1 inhibits both basal and ISO-induced cell migration. n = 4.

We have previously demonstrated that Epac increases melanoma cell migration by modification of heparan sulfate [17]. In addition, since it has been reported that Gβγ plays a role in cell migration of endothelial cells and breast cancer cells [4-6], we examined the effects of Gβγ on melanoma cell migration. We found that mSIRK, a cell-membrane permeable activator of Gβγ [7], decreased basal cell migration only at the highest dose (50 μM). In contrast, mSIRK inhibited Epac1 overexpression-induced cell migration in a dose-dependent manner (Figure 1a). These data suggest that the inhibitory effect of Gβγ is obvious under Epac-activated conditions, but not clear under basal conditions (Figure 1a). Gp(CH)_2_pp, a constitutively active GTP analogue which dissociates Gβγ from Gα, also inhibited Epac-induced cell migration. In addition, mSIRK inhibited cell migration induced by 8-pMeOPT, an Epac-specific agonist, suggesting that Gβγ also inhibits cell migration induced by endogenous Epac (Figure 1b). Further, we examined the specificity of Gβγ in the inhibition of Epac-induced cell migration. Co-overexpression of Gβ1 and Gγ2 subunits (Figure 1c), which is the major combination of Gβγ [25], inhibited Epac1-induced cell migration (Figure 1e). By contrast, overexpression of βARK-CT (Figure 1d), an inhibiting-peptide for Gβγ [26], abolished the mSIRK's inhibitory effect (Figure 1e). These data suggest that there is cross talk between Gβγ and Epac, which affects melanoma cell migration. Since activation of Epac was achieved by artificial overexpression or the Epac-agonist which does not exist in nature, we tested whether hormonal control of GPCR increases cell migration via Epac. Stimulation of β-adrenergic receptor increased cell migration in melanoma, and it was inhibited by ablation of Epac1 (Figure 1f), suggesting that Epac increases cell migration upon activation of hormone receptors.

### Gβγ inhibits Epac-induced Ca ^2+ ^elevation

**Figure 2:**
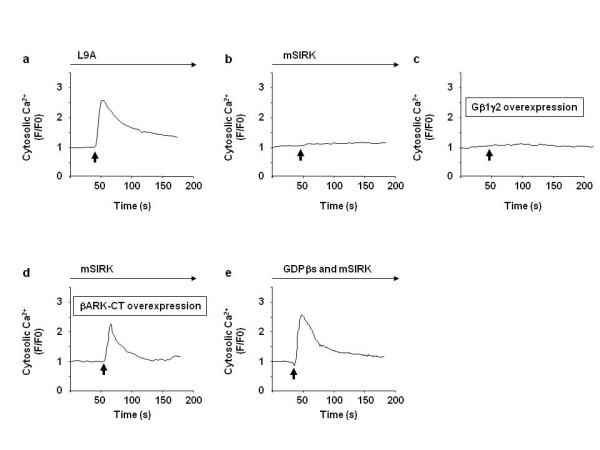
**Gβγ inhibits Epac-induced Ca ^2+ ^elevation**. SK-Mel-2 cells were subjected to Ca ^2+ ^signal measurements. Arrows indicate addition of 8-pMeOPT (200 μM). a and b) SK-Mel-2 cells were pre-incubated with L9A (20 μM) or mSIRK (20 μM) for 5 min followed by the addition of 8-pMeOPT. mSIRK, but not L9A, inhibited 8-pMeOPT-induced Ca ^2+ ^elevation. c) 8-pMeOPT did not increase Ca ^2+ ^signal in SK-Mel-2 cells with co-overexpression of Gβ1 and Gγ2. d) 8-pMeOPT increased Ca ^2+ ^signal in SK-Mel-2 cells with overexpression βARK-CT even in the presence of mSIRK. e) When SK-Mel-2 cells were pretreated with the combination of mSIRK and GDPβs, 8-pMeOPT increased Ca ^2+ ^signal.

We next explored the mechanism by which Gβγ inhibits Epac-induced cell migration. Since our previous report have shown that Epac1-induced cell migration is mediated by Ca ^2+ ^release from the ER [18], we examined whether mSIRK affects Epac1-induced cytosolic Ca ^2+ ^elevation. When cells were pretreated with mSIRK, but not with L9A, 8-pMeOPT failed to increase Ca ^2+ ^signal (Figure 2a and 2b). In addition, co-overexpression of Gβ1 and Gγ2 also inhibited 8-pMeOPT-induced Ca ^2+ ^elevation (Figure 2c). Further, inhibition of Gβγ with βARK-CT, or guanosine 5'-O-(2-thiodiphosphate) (GDPβS), a GDP analogue that inactivates Gβγ, restored 8-pMeOPT-induced Ca ^2+ ^elevation (Figure 2d and 2e). These data suggest that Gβγ inhibits Epac-induced Ca ^2+ ^release.

### Gβγ activates Ca ^2+ ^entry from the extracellular space

**Figure 3:**
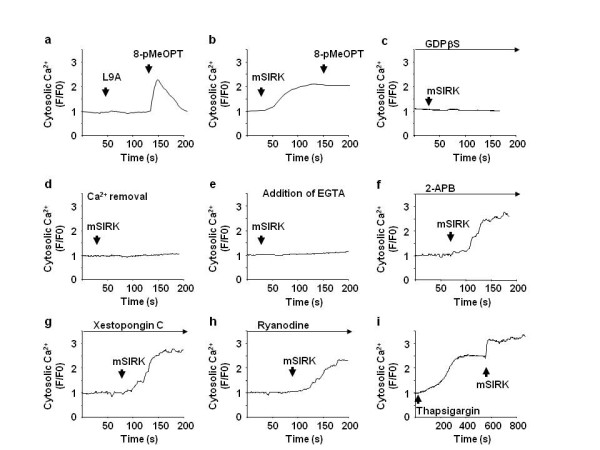
**Gβγ induces Ca ^2+ ^influx from the extracellular space**. SK-Mel-2 cells were subjected to Ca ^2+ ^signal measurements. a and b) SK-Mel-2 cells were incubated with L9A (20 μM) or mSIRK (20 μM) followed by 8-pMeOPT stimulation (200 μM). mSIRK, but not L9A, increased Ca ^2+ ^signal. 8-pMeOPT failed to show an additional increase of Ca ^2+ ^signal after mSIRK. c, d and e) mSIRK-induced Ca ^2+ ^signal was inhibited by pretreatment with GDPβS (100 μM) for 5 min (c), by Ca ^2+ ^removal from the media (d) or by depletion of Ca ^2+ ^in the extracellular space with EGTA (5 mM) (e). SK-Mel-2 cells were subjected to Ca ^2+ ^signal measurements. f, g and h) Inhibition of IP3 receptors with 2-APB (1 μM) (f) or xestopongin C (1 μM) (g), and blocking of ryanodine receptor with ryanodine (10 μM) (h), did not inhibit mSIRK-induced Ca ^2+ ^elevation. i) mSIRK increases Ca ^2+ ^signal SK-Mel-2 cells after depletion of Ca ^2+ ^in the ER with thapsigargin (2 μM).

Since IP3 receptors are inactivated by prior cytosolic Ca ^2+ ^elevation [19-21], we hypothesized that Ca ^2+ ^elevation induced by Gβγ inhibits Epac-induced Ca ^2+ ^elevation. mSIRK, but not L9A, increased Ca ^2+ ^signal in SK-Mel-2 cells (Figure 3a and 3b). mSIRK-induced Ca ^2+ ^elevation similarly observed in various melanoma cells including WM1552C, a regional growth pattern primary melanoma cell line (RGP), WM115, WM1361A, WM3284, vertical growth pattern primary melanoma cells (VGP), and SK-Mel-24, SK-Mel-187 and C8161, metastatic melanoma cell lines (MM) (data not shown), suggesting mSIRK's effect on Ca ^2+ ^is universal in different types of melanoma. After the elevation of Ca ^2+ ^signal by mSIRK, 8-pMeOPT did not show an additional elevation of Ca ^2+ ^signal (Figure 3b). GDPβS inhibited mSIRK-induced cytosolic Ca ^2+ ^elevation, suggesting the specificity of mSIRK for Gβγ (Figure 3c). We next examined the source of Gβγ-induced Ca ^2+^. When extracellular Ca ^2+ ^was depleted by removal of Ca ^2+ ^or by addition of EGTA, a Ca ^2+^-chelating agent, mSIRK did not increase Ca ^2+ ^signal (Figure 3d and 3e, respectively). By contrast, inhibition of IP3 receptors or ryanodine receptor did not affect mSIRK-induced Ca ^2+ ^signal (Figure 3f, g and 3h). Further, depletion of Ca ^2+ ^store in the ER with thapsigargin did not inhibit mSIRK-induced Ca ^2+ ^elevation (Figure 3i). Taken together, these data suggest that Gβγ induces Ca ^2+ ^from the extracellular space, but not from intracellular store.

### Calmodulin is involved in Gβγ-mediated effects on Epac-induced Ca ^2+ ^elevation/cell migration

**Figure 4:**
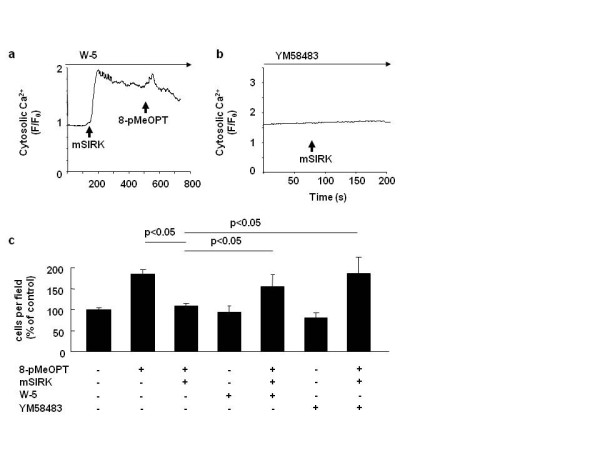
**Calmodulin is involved in Gβγ's inhibition on Epac-induced Ca ^2+ ^elevation, and cell migration**. a) mSIRK (20 μM) and 8-pMeOPT (200 μM) were added in the presence of W-5 (100 μM) in SK-Mel-2 cells. W-5 restored 8-pMeOPT-induced Ca ^2+ ^elevation even in the presence of mSIRK. b) YM58483 (5 μM) inhibited mSIRK-induced Ca ^2+ ^elevation in SK-Mel-2 cells. c) Cell migration assay was performed in SK-Mel-2 cells in the presence or absence of 8-pMeOPT, mSIRK, W-5 or YM58483. W-5 and YM58483 restored Epac-induced cell migration even in the presence of mSIRK. n = 4.

Since IP3 receptor is known to be inactivated by Ca ^2+^/calmodulin [19-21], we examined whether calmodulin mediates Gβγ's inhibitory effect on Epac-induced Ca ^2+ ^elevation. W-5, a calmodulin inhibitor, restored Epac-mediated Ca ^2+ ^signal, and cell migration even in the presence of mSIRK (Figure 4a and 4c, respectively). In order to identify Ca ^2+ ^channel in which Gβγ activates Ca ^2+ ^influx from the extracellular space, we tested Ca ^2+ ^inhibitors in mSIRK-induced Ca ^2+ ^elevation. We then found that, 4-methyl-4' -[3,5-bis(trifluoromethyl)-1H-pyrazol-1-yl]-1,2,3-thiadi azole-5-carboxanilide (YM58483), which is known as an antagonist for Ca ^2+^-activated Ca ^2+ ^channels (CRAC) [27] or canonical transient receptor potential (TRP) channels [28], inhibited mSIRK-induced Ca ^2+ ^signal (Figure 4b). In addition, YM58483 attenuated Gβγ's inhibitory effect on Epac-induced cell migration (Figure 4c). Since YM583483 did not change basal cell migration (Figure 4c), Gβγ-activation is necessary to inhibit Epac-induced cell migration. In contrast, neither verapamil nor nifedipine inhibit mSIRK-induced Ca ^2+ ^(data not shown), suggesting that dihydropyridine Ca ^2+ ^channels are not involved in Gβγ-induced Ca ^2+ ^signal. Put together, Gβγ inhibits Epac-induced Ca ^2+ ^elevation, and cell migration by Ca ^2+^/calmodulin pathway.

## Discussion

**Figure 5:**
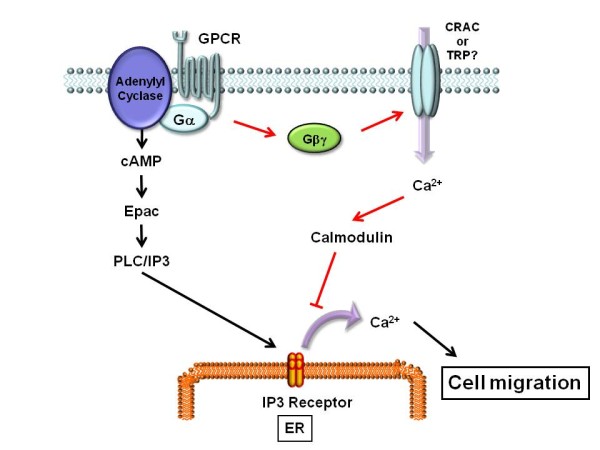
**Ca ^2+ ^signal cross talk between Epac and Gβγ**. Activation of GPCR releases two signaling molecules, Gα and Gβγ. Gα activates cAMP production, leading to Ca ^2+ ^release from IP3 receptor in the ER via Epac/PLC/IP3 pathway. Ca ^2+ ^release from IP3 receptor induces cell migration. On the other hand, Gβγ stimulates Ca ^2+ ^influx from the extracellular space, leading to activation of calmodulin and the following inactivation of IP3 receptor. Finally, Gβγ inhibits Epac-induced Ca ^2+ ^release from the ER, leading to inhibition of cell migration.

In the present study, we demonstrated that Gβγ interferes with the Ca ^2+ ^signaling evoked by Epac, leading to an inhibition of Epac-induced cell migration. We found that Gβγ activates Ca ^2+ ^entry from the extracellular space, which inhibits Epac-induced cytosolic Ca ^2+ ^elevation, and cell migration. Calmodulin is presumably involved in these Gβγ's effects. Since Gβγ is a common molecule located downstream of various GPCRs, our findings would provide novel insights in terms of Ca ^2+ ^signaling, cell migration, and cross talk of intracellular signaling in melanoma (Figure 5).

We previously demonstrated that the expression of Epac1 is increased in metastatic melanoma than in primary melanoma in human melanoma samples. In addition, overexpression of Epac1 enhanced melanoma metastasis in mice [17]. These previous findings indicate that Epac1 accelerates melanoma metastasis, and thus a molecule that can inhibit Epac1 is a potential drug for suppressing melanoma metastasis. The major mechanism by which Epac1 enhances melanoma metastasis is, at least in part, an increase of cell migration via the Ca ^2+^-dependent mechanism [18]. We demonstrated that Epac activates the PLC/IP3 pathway, leading to the activation of IP3 receptor in the ER. Subsequently, Ca ^2+ ^released from the ER modifies actin assembly, and as a result, increases cell migration. In our preliminary study, Epac-induced migration was inhibited by stimulation of different types of GPCRs, i.e., β-adrenergic receptor, lysophosphatidic acid receptor and endothelin receptor. Since types of Gα subunit are different in these GPCRs whereas Gβγ is common in these receptors, we hypothesized that Gβγ mediates the inhibition in Epac-induced cell migration.

Although it is well known that Gβγ regulates membrane Ca ^2+ ^channels in excitable cells, little attention has been paid to its role in non-excitable cells including cancer cells. A report demonstrated that Gβγ activates IP3 receptor via phospholipase C [29]; however, this is not the case in melanoma because antagonists of IP3 receptors did not inhibit mSIRK-induced Ca ^2+ ^elevation (Figure 3f and 3g). Instead, we found that Gβγ induces Ca ^2+ ^from the extracellular space in all melanoma cell lines which we have tested in this study. Moreover, the prior Ca ^2+ ^elevation by Gβγ inhibited Epac-induced Ca ^2+ ^release, suggesting the crosstalk of Ca ^2+ ^signaling between Gβγ and Epac. We also found that calmodulin, an ubiquitously expressed calcium binding protein, presumably mediates this Gβγ's effect (Figure 4a). Since the activity of IP3 receptor is reduced by cytosolic Ca ^2+ ^elevation *via *calmodulin [19-21], and Epac activates IP3 receptor, it is tempting to speculate that calmodulin is activated by Gβγ-induced Ca ^2+^, leading to inactivation of IP3 receptor.

A question remains unclear what Ca ^2+ ^channel(s) is (are) activated by Gβγ. We demonstrated that YM58483 inhibits mSIRK-induced Ca ^2+ ^elevation. YM58483 is known to inhibit CRAC [27]and TRP channels [28], suggesting involvement(s) of these receptors in Gβγ-induced Ca ^2+ ^entry. Further vigorous experiments including knockdown of CRAC or TRP channels are necessary to elucidate the mechanism of Gβγ-mediated Ca ^2+ ^influx. In addition, it is necessary to identify which G-protein coupled receptor(s) is(are) related to Gβγ-induced Ca ^2+ ^entry, and to further investigate whether knocking down of a specific subtype(s) of Gβ or Gγ subunit reduces melanoma cell migration, and metastasis. Meanwhile, mSIRK inhibited Epac-induced cell migration in a dose dependent manner whereas it did not reduce basal cell migration with the exception of the higher concentration (50 μM) (Figure 1a), suggesting that mSIRK's inhibition is obvious only when Epac is activated. This is in accordance with our previous report showing that inhibition of IP3 receptor reduces melanoma cell migration only under Epac-activated conditions [18].

## Conclusion

In summary, the current study demonstrated the potential new cross talk pathway in melanoma in terms of Ca ^2+ ^homeostasis and cell migration. We demonstrated that there is interaction in Ca ^2+ ^signaling between Gβγ and Epac in melanoma. The interaction evoked by Gβγ inhibited Epac-induced Ca ^2+ ^elevation and cell migration via additional Ca ^2+ ^influx from extracellular space. We, thus, propose that Gβγ-signaling inhibits the Epac-induced cell migration via a Ca ^2+^-dependent mechanism in melanoma.

Further studies are needed whether the effects of Gβγ are universal in other non-excitable cells including other types of cancer.

## Abbreviations used

Epac1: exchange protein activated by cAMP; ER: endoplasmic reticulum; IP3: inositol 1,4,5-trisphosphate; Gβγ G-protein βγ subunits; GDPβS: guanosine 5'-O-(2-thiodiphosphate); Gp(CH_2_)pp: guanosine 5', α-β-methylene triphosphate; PKA: protein kinase A; mSIRK: myr-SIRKALNILGYPDYD-OH; L9A: myr-SIRKALNIAGYPDYD-OH; 2-APB: 2-aminoethoxydiphenyl borate; W-5: N-(6-aminohexyl)-1-naphthalenesulfonamide; 8-pMeOPT: 8-(4-Methoxyphenylthio)-2'-O-methyladenosine-3',5'-cyclic monophosphate; βARK-CT: C-terminal domain of β-adrenergic receptor kinase; YM58483: 4-methyl-4'-[3,5-bis(trifluoromethyl)-1H-pyrazol-1-yl]-1,2,3-thiadiazole-5-carboxanilide; CRAC: Ca ^2+ ^release-activated Ca ^2+ ^channels; TRP: transient receptor potential;

## Competing interests

The authors declare that they have no competing interests.

## Authors' contributions

EB designed the study, performed experiments and wrote the manuscript. MU and L-H X performed calcium experiments and participated in the design of the intracellular calcium experiments. MN participated in the design of the study. MSD, MI, SC and JSG participated in writing the manuscript. KI designed the study, performed experiments and wrote the manuscript. All authors read and approved the final manuscript.

## Pre-publication history

The pre-publication history for this paper can be accessed here:

http://www.biomedcentral.com/1471-2407/11/256/prepub
